# Synthesis of 1-(2-Hydroxyphenyl) Dec-2-en-1-One Oxime and Its Flotation and Adsorption Behavior for Malachite

**DOI:** 10.3389/fchem.2020.592771

**Published:** 2020-11-26

**Authors:** Liqing Li, Lin Yang, Fangxu Li

**Affiliations:** ^1^Faculty of Materials Metallurgy and Chemistry, Jiangxi University of Science and Technology, Ganzhou, China; ^2^Institute of Resources Comprehensive Utilization, Guangdong Academy of Science, Guangzhou, China

**Keywords:** collector, flotation, adsorption, malachite, 1-(2-hydroxyphenyl) dec-2-en-1-one oxime

## Abstract

A novel collector of 1-(2-hydroxyphenyl) dec-2-en-1-one oxime (HPDO) was synthesized from 2-hydroxy acetophenone and octanal, and its flotation and adsorption behavior for malachite were studied by flotation tests and x-ray photoelectron spectroscopy (XPS) analysis. The flotation results of a single mineral show HPDO is a special collector for malachite. Compared with benzohydroxamic acid (BHA), isobutyl xanthate (SIBX), and dodecylamine (DA), HPDO exhibits excellent flotation performance for malachite and satisfied selectivity against quartz and calcite over a wide pH range. The HPDO with a concentration of 200 mg/L can float 94% malachite at pH 8, while only recovering 7.8% quartz and 28% calcite. XPS data give clear evidence for the formation of a Cu-oxime complex on malachite surfaces after HPDO adsorption.

## Introduction

Copper was one of the first metals utilized by humans and had a profound impact on the progress of early human civilization. Copper is still an important material today and is widely used in the electrical and electronic markets, transportation, industrial machinery and equipment, organic chemistry, and medicine (Rao et al., 2015; Hussain et al., 2018; Snoussi et al., 2018; Aslam et al., 2019). Copper demand is expected to reach 26.4 million tons by 2020 (Elshkaki et al., 2016). The amount of copper in the earth's crust is about 0.01%, and native copper is rare in nature, found mostly in the form of copper ore. More than 170 copper-containing minerals have already been found. However, only 10–15 of these minerals have been successfully developed commercially. Because the mineral process often brings environmental pollution problems, it is very important to apply copper resources reasonably and efficiently (Chan et al., 2014; Li et al., 2019b). The most common copper sulfide includes chalcopyrite, bornite, and beryllium copper ore. With the further exploitation of known copper sulfide ores, copper oxide ore can serve as a new source of copper to meet the huge market demand (Huang et al., 2018; Wang et al., 2020).

Malachite is one of the most common copper oxide ores. Leaching-SX (solvent extraction)-EW (electrowinning) is a commercial method used for processing copper oxide ores (Li et al., 2015). While this process has shortcomings, such as its lengthy processing steps, high agent consumption, and high extractant cost (Liu et al., 2016) its flotation of malachite can overcome the above disadvantages and gradually become a hotspot for flotation scientists and engineers (Marion et al., 2017). Recently, two methods of sulfide flotation and direct flotation were used in the flotation of malachite. As for sulfide flotation, traditional copper sulfide flotation collectors do not perform well with hydrophilic malachite, unless it is activated by Na_2_S, NaHS, (NH_4_)_2_S, NH_4_HS, 8-hydroxyquinoline or diethylamine phosphate (Shengo et al., 2014; Cui et al., 2015; Park et al., 2016; Feng et al., 2018a,b; Qin et al., 2018). In addition, sulphidisation for copper oxide ore was hard to control because of the complex ore properties (Corin et al., 2017). As for direct flotation, fatty acids, fatty amines, sulfonates, and phosphonates reveal poor selectivity in separating malachite from calcite (Li et al., 2015, 2018). Hydroxamic acids, such as BA and Octyl hydroxamic acid, show a satisfying flotation performance for malachite (Hope et al., 2012; Mao et al., 2014). However, oxime collectors have not received enough attention over the past two decades.

Oxime, which is in the C=NH-OH group, is similar to hydroxamic surfactants and has proven to be an impressive collector for malachite in minerals engineering. Salicylaldoxime has a strong affinity to Cu(II), and is widely studied in copper oxide ore. Li found the recovery of malachite reached 97% by using salicylaldoxime as a collector with the help of 10^−2^ mol/L Na_2_CO_3_ (Li et al., 2019). Han used a combination collector of salicylaldoxime and xanthate to deal with complex copper oxide ores from the Yulong copper mine in China. However, the synergy mechanism of salicylaldoxime and xanthate has not yet received the attention of researchers (Han et al., 2017; Wang et al., 2019). Li found that Tert-butyl salicylaldoxime is a powerful collector for malachite (Li et al., 2019a). 2-hydroxy-5-nonylphenyl (phenyl) methanone oxime has proven to be proficient at the beneficiation of copper oxide ore (Yang et al., 2011). The other oximes, such as 2-ethyl-2-hexenaloxime (Xu et al., 2014), are effective collectors for malachite or chalcopyrite. However, 1-(2-hydroxyphenyl) dec-2-en-1-one oxime (HPDO) for the recovery of copper oxide ore has been hardly studied.

The aim of this paper is to provide a reference for the flotation performance adjustment through efficient molecular structure modification. A novel ketoxime collector was synthesized through aldol condensation and ammoximation reaction. The chemical structure of HPDO is characterized by FTIR and LC-MS. The flotation performance of HPDO to malachite was studied by micro and batch flotation tests. And the adsorption mechanism of HPDO on malachite was discussed by XPS analysis.

## Experimental

### Materials

**Figure 1:**
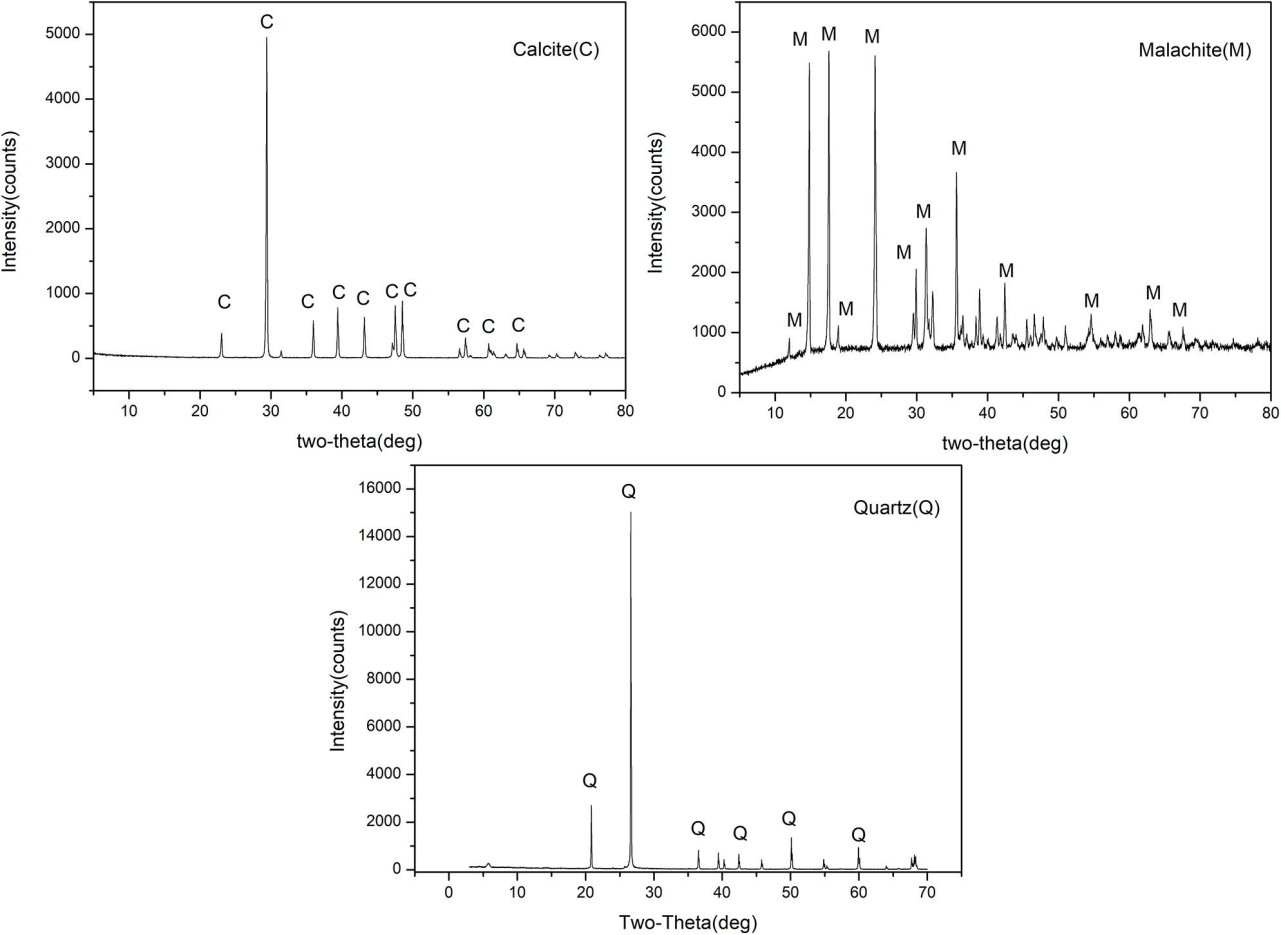
XRD of malachite, quartz, and calcite.

The XRD of malachite, quartz, and calcite were shown in Figure 1. The element analysis of malachite, quartz, and calcite were listed in Table 1. −75 + 38 μm fractions of single minerals were utilized in micro-flotation experiments, and −5 μm fractions were prepared for XPS measurement. Copper oxide ore from Jiangxi province in China was used in batch flotation. The most abundant copper oxide mineral was malachite (1.24%). The major gangue minerals in the ore were quartz (70.68%) and calcite (24.31%). BHA and SIBX were obtained from Zhuzhou Flotation Reagents & Chemicals Co., Ltd in China. DA was purchased in Aladdin Reagent Company. HPDO was synthesized in our laboratory. Before flotation, collectors were dispersed by NaOH or HCl aqueous solution through ultrasound.

**Table 1:** The compositions of malachite, calcite, and quartz samples analyzed by element analysis.

**Composition (wt%)**	**CuO**	**CaO**	**SiO_**2**_**	**CO_**2**_**	**Others**
Malachite	70.53	–	0.19	19.58	9.7
Quartz	–	–	98.12	–	1.88
Calcite	–	53.90	–	42.14	3.96

### Flotation

**Figure 2:**
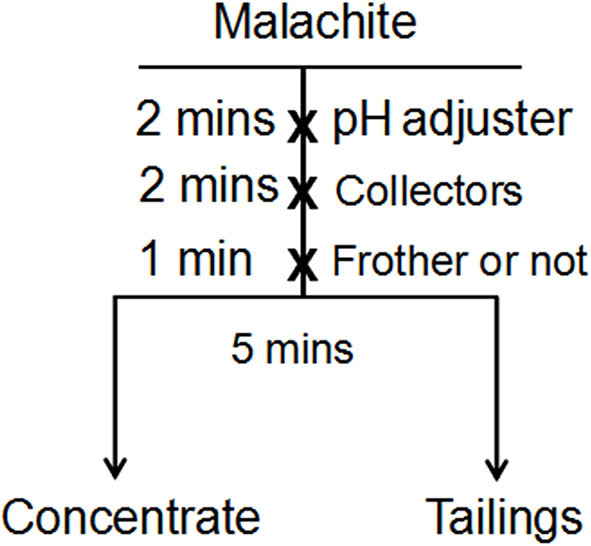
The flowstep of the micro-flotation experiment.

A micro-flotation experiment was implemented on a flotation machine (Jilin Province Prospecting Machinery Factory) with an effective cell volume of 40 mL. First, a single mineral was dispersed in water for 2 min. Secondly, Na_2_CO_3_ (1%) or HCl (1%) were introduced to adjust the pH of the pulp. Next, a collector was added for 2 min. Fourthly, the frother was conditioned for 1 min. Finally, the conditioned slurry was floated for 5 min. The flotation results of the single mineral were calculated from the dry weights of the concentrates and tails. The flowstep of the micro-flotation experiment is shown in Figure 2.

**Figure 3:**
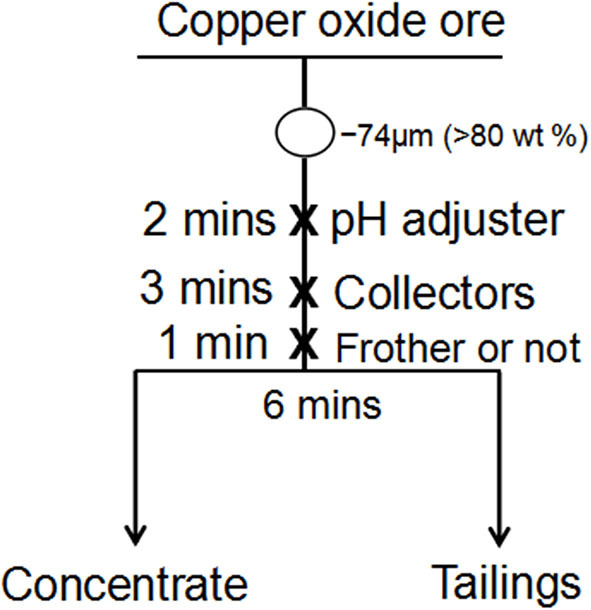
The flowstep of the batch-flotation experiment.

A batch-flotation test was conducted by operating a flotation machine (Jilin Province Prospecting Machinery Factory) with an effective cell volume of 750 mL. First, the copper oxide ore was ground to −74μm (>80 wt%) in an XMB-70 rod mill. After that, the pH of the copper oxide ore for batch flotation was adjusted at around 8.0. Then, the collector was added for 3 min. The conditioned slurry was floated for 6 min. Finally, the concentrates and tailings were dried and weighed, and the amount of Cu was detected through chemical analyses. The flowstep of the batch-flotation experiment is shown in Figure 3.

### XPS Analysis

XPS analyses were operated on a Thermo Scientific ESCALAB 250 Xi instrument using an Al Kα X-ray source; the condition was 200 W with pass energy of 20 eV. All samples were tested under vacuum pressure. In order to reveal the adsorption mechanism, the data of malachite before and after HPDO treatment were carefully collected and treated by using Thermo Scientific Advantage 4.52 software.

## Results and Discussion

### Preparation and Characterization of HPDO

**Figure 4:**
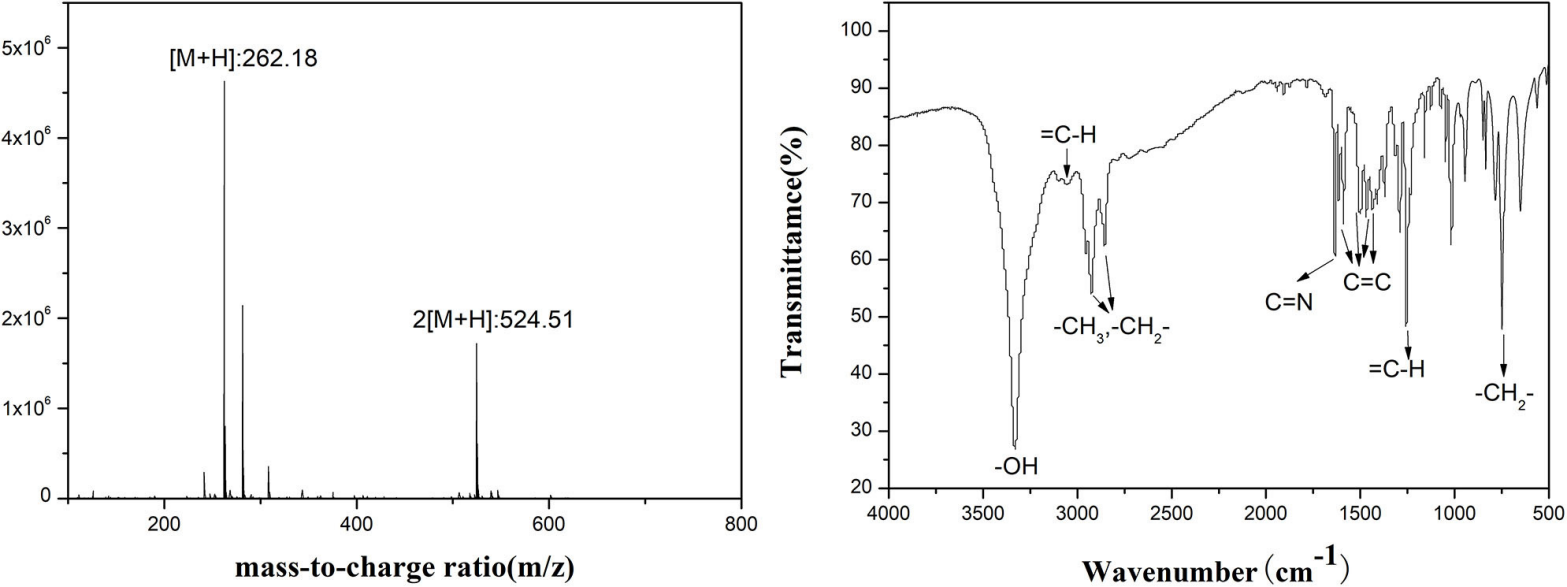
FTIR and ESI-MS of HPDO.

HPDO was prepared by using 2-hydroxy acetophenone, octanal, and NH_2_OH·HCl as the starting materials. First, 2-hydroxy acetophenone and octanal were added to methanol (50 mL) using 10% NaOH (5 mL) as a catalyst and stirred at 60°C for 6 h. Next, NH_2_OH·HCl (7.65 g, 0.11 mol) in H_2_O (50 mL) were added to a mixture of 1-(2-hydroxyphenyl) dec-2-en-1-one. After this, NaOH solution (5%) was added to adjust the pH range of the solution to 7–8 and reacted for 4 h at 50°C. Finally, the brown crude mixture was obtained after removing the solvent. Product characterization: 1-(2-hydroxyphenyl) dec-2-en-1-one oxime (HPDO), yield of 56.35%. IR (film, cm^−1^): 3,328 (-OH), 3,056 (aromatic C-H), 2,925 and 2,854 (CH_3_- and -CH_2_-), 1,628 (C=N), 1,607, 1,587, 1,501, and 1,466 (aromatic C-H), 1,254 (H-C=C), 750 (-CH_2_-). ESI-MS (ESI^+^): calculated for C_16_H_23_NO_2_ 261.17; found 262.18 [M + H] (See Figure 4).

### Flotation Tests

**Figure 5:**
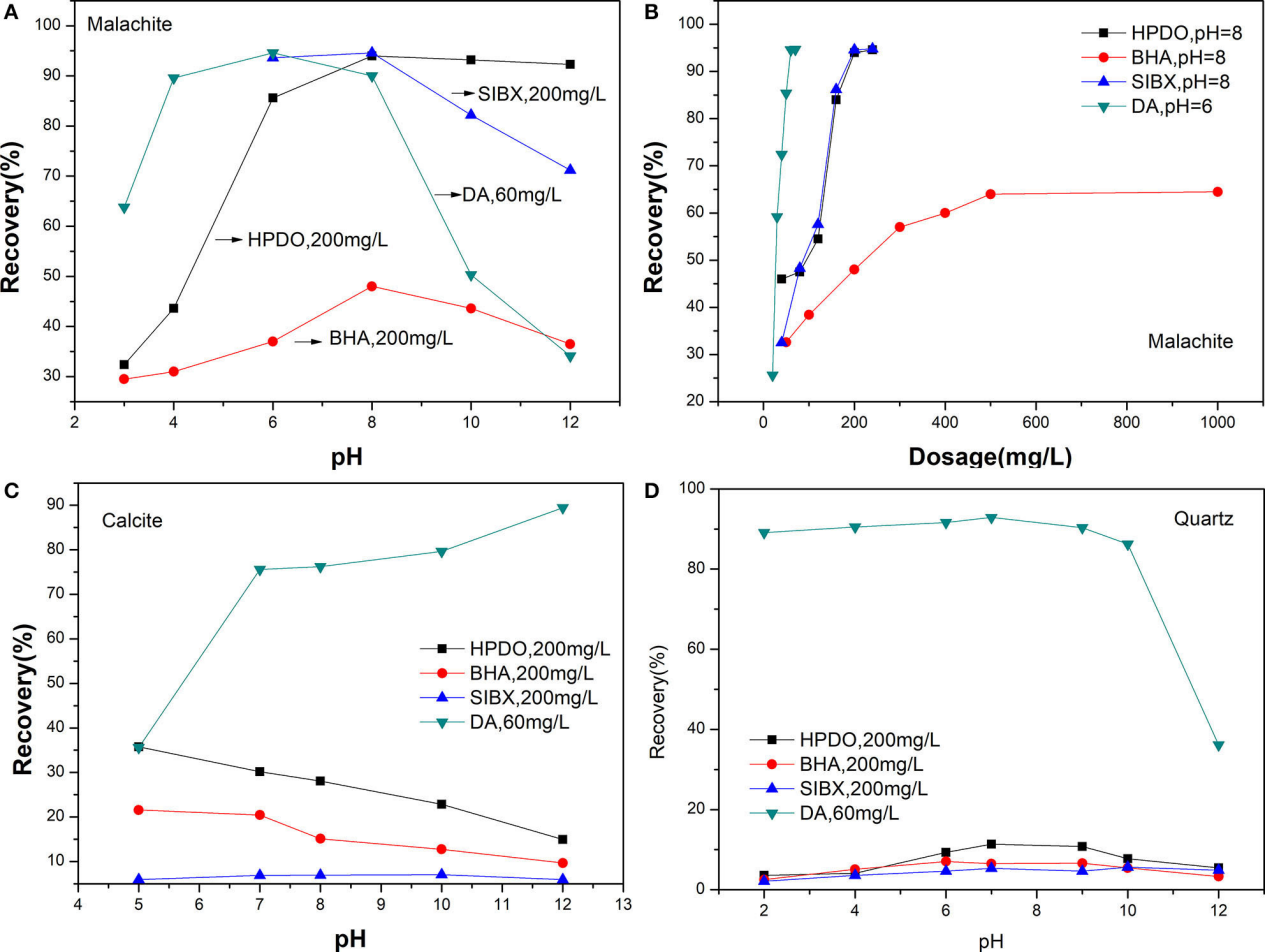
**(A)** Flotation recovery of malachite with different collectors. **(B)** The effect of the collector dosage on the flotation recovery of malachite. **(C)** The effects of pH values on the flotation recoveries of quartz. **(D)** The effects of pH values on the flotation recoveries of calcite.

The flotation results of malachite, quartz, and calcite by four collectors are shown in Figure 5. The effect of pH on the flotation recovery of malachite is given in Figure 5A. It shows that the best flotation results for HPDO, BHA, SIBX, and DA are obtained at a pH of 8, 8, 8, and 6, respectively. The maximum recovery of malachite with HPDO, SIBX, and DA are close to 95%, but the maximum recovery of malachite with BHA is just 48.0%. When the pH is <8, the recovery of malachite gradually increased by using HPDO, BHA, and SIBX as collectors. When the pH is more than 8, the recovery of malachite decreased swiftly, except with HPDO. These results indicate that HPDO has wider flotation pH values for malachite than BHA, SIBX, or DA. The effect of the collector dosage on the flotation recovery of malachite is shown in Figure 5B. The result of HPDO shows that the flotation recovery increases rapidly at an initial dosage ranging from 40 to 200 mg/L and almost reaches maximum (94%), and when the dosage of HPDO is more than 200 mg/L, the recovery slowly increases. SIBX shows a similar flotation performance to HPDO. As for DA, the recovery increases swiftly at low dosage, and DA can recover 94.6% malachite at a dosage of 70 mg/L. When the dosage of BHA is 200 mg/L, its recovery of malachite is just 48%. Even if the dosage of BHA is up to 1,000 mg/L, its recovery is just up to 65%. The effects of pH values on the flotation recoveries of quartz and calcite are listed in Figures 5C,D. It is clear from Figures 5C,D that HPDO, BHA, and SIBX show good selectivity to quartz or calcite, and those recoveries are <40%. While for DA, the recovery of calcite and quartz are more than 70% at a wide pH; it indicates that DA is not a good choice for a Cu-Ca-Si flotation system.

Micro-flotation tests showed that HPDO is a powerful collector for malachite, and achieves a satisfactory selectivity against quartz. While DA lacks selectivity to calcite and quart; SIBX needs additional Na_2_S and foaming agent added; BHA has a weak collecting power. Compared with BHA, SIBX, and DA, HPDO exhibited an excellent collecting power for malachite without an additional reagent. This seems to indicate that our method of introducing a long carbon chain into a precursor with chelating ability is effective.

The batch flotation results of malachite with collectors are shown in Table 2. When the dosage of HPDO is 300 g/t through single roughing flow, the copper concentrate grade and recovery are 4.38% and 66.64%, respectively. With BHA (300 g/t), SIBX (300 g/t), or DA (100 g/t), the copper concentrate grade and recovery are not satisfied (detailed in Table 2). BHA and SIBX show poor recovery, and <35% copper is obtained. DA shows bad selectivity to Jiangxi copper oxide. It seems that HPDO has the potential to be used in flotation of copper oxide ores.

**Table 2:** Flotation results of copper oxide with different collectors.

**Collectors**	**Dosage (g/t)**	**MIBC (g/t)**	**Na2S (g/t)**	**Na2SiO3 (g/t)**	**Recovery (%)**	**Grade Cu (%)**
HPDO	300	–	–	800	66.64	4.38
BHA	300	40	–	800	15.90	2.44
SIBX	300	40	200	800	34.26	3.25
DA	100	–	–	800	71.12	0.98

### XPS Analyses of Malachite Before and After HPDO Adsorption

**Figure 6:**
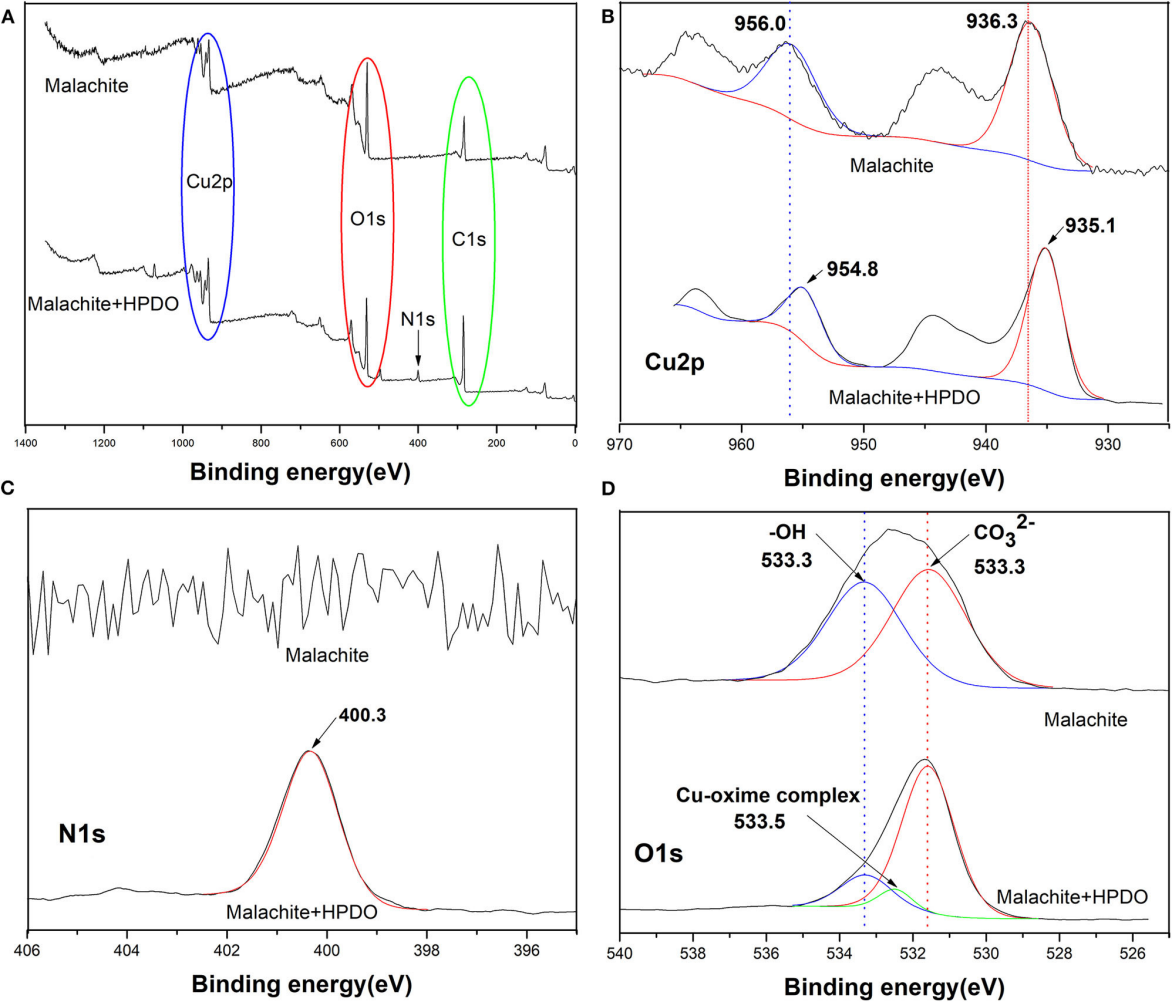
**(A)** XPS spectra of malachite before and after HPDO adsorption. **(B)** XPS spectra of Cu 2p. **(C)** XPS spectra of N1s. **(D)** XPS spectra of O1s.

XPS was used to check the changes of surface compositions and atoms of malachite with or without HPDO treatment. The survey XPS spectra of malachite before and after HPDO modification are shown in Figure 6A. The relative changes in the elements on the malachite surface can prove whether the mineral surface is covered by collectors. It is obvious that the relative intensity of C1s and N1s increase after HPDO adsorption, while the relative intensity of O1s decreases. It indicates that the malachite surface had been coated by numerous HPDOs with a high concentration of O element. The nitrogen signal observed in Figure 6A is consistent with the flotation result of malachite in the HPDO solution.

In order to get more data for HPDO adsorption on a malachite surface, it is important to verify that high-resolution XPS spectra of N1s, O1s, and Cu2p before and after HPDO modification (Yumitori, 2000). Compared with malachite without any treatment, the binding energy of Cu2p3/2 and Cu2p1/2 of malachite-HPHO products both decreased by 1.2 eV (See Figure 6B). It hinted that copper atoms received electron form electronegative HPDO. As can be seen in Figure 6C), the binding energy of N1s stands around 400.3 eV after HPDO adsorption on the malachite surface. The N1s signal offered clear evidence that HPDO reacted with Cu species on malachite. The inspection of the O1s XPS is very important to examine the chemical changes on malachite after HPDO modification (See Figure 6D). The decrease of the relative peak ratio of -OH with respect to $CO_{3}^{2-}$ after HPDO treatment indicated the OH groups on malachite were replaced and coupled by HPDO, which was consistent with the analysis of Cu2p.

## Conclusion

(1) HPDO was synthesized through aldol condensation and ammoximation reaction.
(2) The flotation results indicated that HPHO was a special collector for malachite flotation, whose collecting ability is improved as we desired. This indicates that HPDO has the potential to deal with low-grade copper ores in minerals engineering.
(3) XPS results offer clear evidence that OH groups on malachite surfaces were replaced and coupled by HPHO, and Cu(II) species was bound with HPDO.
(4) In the future, the solution chemistry of HPDO and the changes in hydrophilicity and hydrophobicity of malachite before and after HPDO, and adsorption thermodynamics, will be more thoroughly studied.

## Data Availability Statement

All datasets presented in this study are included in the article/supplementary material.

## Author Contributions

LL performed the experiments, analyzed all the data, drafted all the figures, and prepared the manuscript. LY performed the experiments. FL revised the manuscript. All authors contributed to the article and approved the submitted version.

## Conflict of Interest

The authors declare that the research was conducted in the absence of any commercial or financial relationships that could be construed as a potential conflict of interest.
